# Editors-in-chief perceptions of patients as (co) authors on publications and the acceptability of ICMJE authorship criteria: a cross-sectional survey

**DOI:** 10.1186/s40900-021-00290-1

**Published:** 2021-06-14

**Authors:** Kelly D. Cobey, Zarah Monfaredi, Evelyn Poole, Laurie Proulx, Dean Fergusson, David Moher

**Affiliations:** 1 Clinical Epidemiology Program, Ottawa Hospital Research Institute, Ottawa, K1H 8L6 Canada; 2 School of Epidemiology and Public Health, Faculty of Medicine, University of Ottawa, Ottawa, K1G 5Z3 Canada; 3 Life Sciences, Faculty of Arts & Science, Queen’s University, Kingston, K7L 3N6 Canada

**Keywords:** Authorship, Publication ethics, Publication best practices, Patient involvement, Authorship criteria, Authorship policy

## Abstract

**Background:**

Access to, and awareness of, appropriate authorship criteria is an important right for patient partners. Our objective was to measure medical journal Editors-in-Chief’ perceptions of including patients as (co-)authors on research publications and to measure their views on the application of the ICMJE (International Committee of Medical Journals Editors) authorship criteria to patient partners.

**Methods:**

We conducted a cross-sectional survey co-developed with a patient partner. Editors-in-Chief of English-language medical journals were identified via a random sample of journals obtained from the Scopus source list. The key outcome measures were whether Editors-in-Chief believed: 1) patient partners should be (co-)authors and; 2) whether they felt the ICMJE criteria for authorship required modification for use with patient partners. We also measured Editors-in-Chief description of how their journal’s operations incorporate patient partner perspectives.

**Results:**

One hundred twelve Editors-in-Chief responded to our survey (18.7% response rate; 66.69% male). Participants were able to skip any questions they did not want to answer, so there is missing data for some items. 69.2% (*N* = 74) of Editors-in-Chief indicated it was acceptable for patient partners to be authors or co-authors on published biomedical research articles, with the remaining 30.8% (*N* = 33) indicating this would not be appropriate. When asked specifically about the ICMJE authorship criteria, and whether this should be revised to be more inclusive of patient partners, 35.8% (*N* = 39) indicated it should be revised, 35.8% (*N* = 39) indicated it should not be revised, and 28.4% (*N* = 31) were unsure about a revision. 74.1% (*N* = 80) of Editors-in-Chief did not think patients should be required to have an academic affiliation to published while 16.7% (*N* = 18) and 9.3% (*N* = 10) indicated they should or were unsure. 3.6% (*N* = 4) of Editors-in-Chief indicated their journal had a policy that specifies how patients or patient partners should be considered as authors.

**Conclusions:**

Our findings highlight gaps that may act as barriers to patient partner participation in research. A key implication is the need for education and for consensus building within the biomedical community to establish processes that will facilitate equitable patient partners inclusion.

**Supplementary Information:**

The online version contains supplementary material available at 10.1186/s40900-021-00290-1.

## Plain English summary

In academia, authorship on a research publication is a central means to obtain credit for one’s contribution to a research project. In order to guide authorship decision making and facilitate transparent processes, the International Committee of Medical Journal Editors (ICMJE) has produced recommendations for authorship. These recommendations are widely adopted by hundreds of medical journals. However, there is no research on whether the ICJME’s recommendations for authorship appropriately recognize the distinct contributions of patient partners to research. This survey study asked Editors-in-Chief about their perceptions of patient partners as authors and on the suitability of the ICMJE authorship criteria. We find that a 30.8%of medical journal Editors-in-chief do not view the inclusion of patient partners as authors on manuscripts as appropriate. Editors-in-chief reported wide ranging views on whether the ICMJE criteria for authorship were appropriate for patient partners. The implication of this work is that there is a need for education and for consensus building within the biomedical community to establish processes that will facilitate patient partners equitable inclusion in research, including on research outputs like publications.

**Supplementary Information:**

The online version contains supplementary material available at 10.1186/s40900-021-00290-1.

## Introduction

There is growing recognition that involving patients as team members in the design and conduct of research adds value [1]. We define patients according to the Canadian Institutes of Health Research’s Strategy for Patient Oriented Research’s (SPOR) definition: “*An overarching term inclusive of individuals with personal experience of a health issue and informal caregivers, including family and friends*” [2]. Biomedical researchers and patients often identify different priorities related to research. A review of studies on kidney dialysis illustrates why this matters: four of the top ten research priorities identified by patients received virtually no attention in the literature. While researchers placed their focus on hemodialysis, patients placed more concern on factors like symptoms experienced (e.g., itchiness) and on assessment of psychosocial impact of kidney failure [3]. Patient-oriented research, in which patients are engaged as members of the research team across the continuum of the research process [4], provides biomedical research with a broader perspective and acknowledges that those with lived experience are well-positioned to identify health care needs and factors that would improve their quality of life [5, 6]. Failure to conduct patient-oriented research risks conducting research that is not relevant or feasible to the patient population. The value of patient-oriented research is recognized in several different jurisdictions. Within Canada, the Canadian Institutes of Health Research created its Strategy for Patient-Oriented Research in 2011 [4]. Similar initiatives were well underway by this time in other countries including the Patient-Centered Outcomes Research Institute (PCORI) in the USA [7] and involve in the United Kingdom [8]. Other stakeholders, including biomedical journals, have also begun incorporating patient perspectives into their operations. For example, the British Medical Journal (BMJ) started a patient and public peer review program in 2014. These types of peer review programs are unfortunately rare. It is encouraging to see that other journals, including Research Involvement and Engagement, have since adopted this practice.

Meaningful engagement of patients as research partners comes with challenges [9–11]. Patient partner involvement can include, but is not limited to, involvement in planning, priority-setting, conducting, disseminating, or translating research findings. It involves a significant culture shift in the way research is conducted. We examine how authorship on research publications may need to be reconsidered to better support patient partnership and inclusion. Publishing research findings in academic journals is the primary venue through which findings are shared in the research community, and authorship on publications confers credit to those who conducted and contributed to the work. Authorship, rightly or wrongly, is at the heart of the academic reward system, often considered the currency for success, with a pressure to publish and its negative consequences well recognized [12, 13]. The question of who to list as authors of a publication can attract controversy and conflict among team members. In medicine, the International Committee of Medical Journal Editors (ICMJE) provides the most widely implemented criteria for authorship. The ICMJE first proposed their criteria in 1985, with subsequent updates released and hundreds of biomedical journals globally embracing the requirements [14]. The ICMJE does not presently provide recommendations for how patient partners should be involved as authors on publications.

As patient engagement in research grows, it seems appropriate to include patients as authors and owners of the research to which they contribute. Authorship on publications serves to recognize patient partner contributions in a way that identifies them as true partners within the overall research project. However, in the absence of ICMJE criteria for authorship that apply to patient partners, it is unclear how journals perceive or handle manuscripts with patient partners named as (co-)authors. The key aims of this study were to: 1) measure the medical journal Editors-in-Chief’ perceptions of acknowledging patients as (co-)authors on research publications; and 2) measure medical journal Editors-in-Chief views on the application of the ICMJE authorship criteria to patient partners. Addressing these aims is critical to establishing equitable guidance and reducing barriers for patient partners as authors of research publications. We also measured Editors-in-Chief description of how their journal’s operations incorporate patient partner perspectives.

## Methods

### Ethics statement

This study obtained research ethics approval from the Ottawa Health Science Network Research Ethics Board (OHSN-REB #20200464-01H; see https://osf.io/utrwx/). This study protocol was registered prior to data collection, using the Open Science Framework [15] (https://osf.io/cqeg8). A preprint of the work is available on the Open Science Framework (https://osf.io/n4rg6/).

### Patient and public involvement

This study was co-designed and produced with a patient partner (LP). They were involved in the development of the research question, drafting of the study protocol, design of the survey, and with the revision of the manuscript (see GRIPP 2 Checklist in Additional file 1).

### Study design

We conducted a cross-sectional survey of Editors-in-Chief of biomedical journals. The survey was voluntary and completed anonymously.

### Journal sampling

We identified potential participants through a random sampling of 600 biomedical journals. We used the Scopus source list (searched on June 15th, 2020), which contains all MEDLINE and EMBASE content to derive our sample. We restricted the Scopus source list to journals with an ASJC classification code of ‘Medicine’ and that were listed as active. We excluded all journals that specified they published exclusively in a language other than English. Then, we sorted the list into two groups: 1) journals that are in the DOAJ (Directory of Open Access Journals); and 2) journals that are not in the DOAJ. Using Excel, we generated a random number table to randomly select 300 journals from each of these two groups. This allowed us to equally sample traditional subscription-based journals as well as open access journals. We replaced any journals that were not available online, or that were no longer publishing, with another randomly selected journal. The sample size of 600 journals was selected for feasibility purposes.

### Identifying participants

We identified the Editors-in-Chief of the selected journals through searching their journal websites. A team member (EP, ZM) extracted the Editor-in-Chief’s e-mail address from each journal website. In cases where an Editor-in-Chief was listed without a corresponding e-mail on the journal website, we Google searched for the Editor-in-Chief using their name and listed affiliation(s), if available. We searched the first five hits on Google to obtain their e-mail. If we were unable to locate their e-mail, we searched the journal website for another relevant contact email. If this was not available or was overly generic and unlikely to reach the Editor-in-Chief, we excluded the journal and replaced it with another randomly selected title from the same journal type (e.g., subscription-based or open access) list. If more than one Editor-in-Chief was listed at a given journal, the first person listed was extracted. In instances where the Editor-in-Chief was not listed, we recorded this, then replaced the journal with another randomly selected title from the same journal list. We used the extracted e-mails of Editors-in-Chief to share a survey. To do so, we sent a standard recruitment script (Appendix 1). E-mails were sent using Mail Merge software. We recorded when e-mails bounced back and replaced these with new journals.

### Survey

Participants were presented with an online consent form that described the aims of the study, specified data collected would be anonymously, and detailed our data management plan which includes making data openly available. Participants were invited to complete a short online survey administered using SurveyMonkey software [16]. After providing informed consent, they were presented with an 18-item survey (see Appendix 2). This survey was co-developed with a patient partner (LP) and piloted for clarity and whether it is fit for purpose by an editor not involved in the study. Participants were able to skip any items they did not want to respond to. Participants were given 3 weeks to complete the survey, with reminders sent to all participants after 1 and 2 weeks from the initial invite to take part, respectively.

### Analyses

Descriptive statistics (counts, percentages) were calculated for all quantitative items on the survey using SPSS 27. We report the overall sample size for each item, and whether data was missing or not. For survey items which required a text-based response we used inductive content analysis. To do so, two reviewers (KDC, ZM) familiarized themselves with the data and then independently generated initial codes for unique themes for each question. They then reviewed the codes collaboratively to search for themes and redefined these where appropriate. We conducted a series of chi-squared tests to examine potential differences between responses to the authorship items (items 10–16 in Appendix 2) based on journal type.

## Results

The STROBE [17] and CHERRIES [18] reporting guideline were used to guide reporting of our findings. STROBE and CHEERIES are reporting guideline checklists of observational studies and online surveys, respectively. Use of reporting guidelines helps to ensure the completeness of reporting of essential study information. Data has been shared using the Open Science Framework [15] (https://osf.io/n9g28/.).

### Participants

A total of 112 Editors-in-Chief responded to our survey (18.7% response rate). Demographics of participants are summarized in Table 1. 75 (67.0%) of participants indicated they were male, 35 (31.3%) were female, and 2 (1.8%) preferred not to indicate their gender. There was considerable range in the length of time participants had been editor of their current journal (less than 1 year to > 6 years), with the majority holding their position for more than 6 years (*N* = 42, 37.5%).

**Table 1:** Participant demographics

	N (%)
Gender (*N* = 112)	
Female	35 (31.25)
Male	75 (66.69)
Other	0 (0)
Prefer not to say	2 (1.79)
Age (*N* = 112)	
Under 26	0 (0)
26–35	0 (0)
36–45	15 (13.39)
46–55	28 (25.00)
56–65	37 (33.04)
Over 65	32 (28.57)
How long have you served as the Editor-in-Chief of your journal? (*N* = 112)	
Less than 1 year	9 (8.04)
1–2 years	22 (19.64)
3–4 years	24 (21.43)
5–6 years	15 (13.39)
More than 6 years	42 (37.50)

### Descriptive journal data

Descriptive information about the journals are summarized in Table 2. Briefly, 63 (56.8%) of the journals participating Editors-in-Chief represented were open access journals while 48 (43.2%) were subscription-based journals. Most Editors-in-Chief reported to be representing a journal that published ‘primarily clinical research’ (*N* = 39, 35.1%) or a combination of both clinical research and preclinical research (*N* = 38, 34.2%). Most Editors-in-Chief reported to work at a clinical specialty journal (*N* = 56, 50.5%) and reported their journal had an impact factor less than 2 (*N* = 50, 45.1%). There were a diverse range of journal locations, with journals from the United States (*N* = 34, 32.1%), the United Kingdom (*N* = 22, 20.8%), and India (*N* = 6, 5.7%), comprising the top three journal locations indicated.

**Table 2:** Journal demographics

	N (%)
Is your journal fully open access (e.g., all articles are immediately published in a format that makes them free to use and free to build upon to all readers, for example, through Creative Commons licensing)? (*N* = 111)	
Yes	63 (56.76)
No	48 (43.24)
Unsure	0 (0)
What is the current impact factor of your journal?	
Less than 2.0	50 (45.05)
2.0–4.0	29 (26.13)
5.0–8.0	5 (4.50)
8.0–15.0	1 (0.90)
More than 15.0	1 (0.90)
The journal does not have an impact factor	21 (18.92)
Unsure	4 (3.60)
What type of research does your journal publish? (*N* = 111)	
Primarily clinical research	39 (35.14)
Primarily basic or pre-clinical research	10 (9.01)
A combination of both clinical research and pre-clinical research	38 (34.23)
Other	24 (21.62)
What area best describes the type of research your journal publishes? (*N* = 111)	
General medical journal	13 (11.71)
A research methods medical journal (e.g., systematic reviews journal)	5 (4.50)
A clinical specialty journal (e.g., Cancer research)	56 (50.45)
Other	37 (33.33)
Top 3 countries where journals were based (*N* = 106)	
United States of America	34 (32.1)
United Kingdom	22 (20.8)
India	6 (5.7)
Other	44 (41.51)

### Editors-in-chief perceptions of patient partner authorship and ICMJE authorship criteria

Main outcome data is summarized in Table 3. A total of 69.2% (*N* = 74) of Editors-in-Chief indicated that it was acceptable for patient partners to be authors or co-authors on published biomedical research articles, with the remaining 30.8% (*N* = 33) indicating this would not be appropriate. Seventy-one (63.4%) participants provided written feedback and their responses were thematically grouped into eight categories. Table 4 provides a summary of these thematic groups.

**Table 3:** Editors-in-Chief perceptions of appropriateness of ICMJE authorship criteria in relation to patient partnership

		N (%)
In your view, is it appropriate for patient partners to be authors or co-authors on published biomedical research articles? (*N* = 107)		
Yes		74 (69.2)
No		33 (30.8)
In your view, do you think the ICMJE authorship criteria should be revised to be more inclusive to patient partners? (*N* = 109)		
Yes		39 (35.8)
No		39 (35.8)
Unsure		31 (28.4)
In your view, which criteria of the ICMJE authorship criteria would a patient partner be likely to meet?		
Substantial contributions to the conception or design of the work; or the acquisition, analysis, or interpretation of data for the work (*N* = 109)	Yes	70 (64.2)
	No	28 (25.7)
	Unsure	11 (10.1)
Drafting the work or revising it critically for important intellectual content (*N* = 109)	Yes	61 (56.0)
	No	34 (31.2)
	Unsure	14 (12.8)
Final approval of the version to be published (*N* = 109)	Yes	74 (67.9)
	No	21 (19.3)
	Unsure	14 (12.8)
Agreement to be accountable for all aspects of the work in ensuring that questions related to the accuracy or integrity of any part of the work are appropriately investigated and resolved (*N* = 108)	Yes	46 (42.6)
	No	39 (36.1)
	Unsure	23 (21.3)
Should patients have to have an academic affiliation to publish as an author/co-author? (*N* = 108)		
Yes		18 (16.7)
No		80 (74.1)
Unsure		10 (9.3)

**Table 4:** Thematic grouping of text-based responses describing why or why not patients should be permitted to be authors or co-authors on biomedical manuscripts

Category	N (%)^a^	Example statements
Contributes to research positively	13 (18.3)	*“It is a relevant action that must be accomplished by the journals”* *“In many cases, they make significant contributions to data acquisition and analysis or perform critical functions, without which, the studies could not be completed”*
Supports equity between patients and researchers	9 (12.7)	*“Their opinions are as worth as those of medical/technical authors”* *“it is a matter of ownership and equity (ie fairness)”*
Statement reflects a misunderstanding of patient partnership	3 (4.2)	*“Patient, who are participating in study are not independent authors and may have inappropriate influence on final form of manuscript”* *“As subjects in the study, their inclusion would likely introduce significant bias.”*
Patients and researchers must follow same authorship rules	18 (25.4)	*“if they meet the criteria for authorship as defined by ICMJE see no problem”* *“Unless the patient was involved in the design and analysis of the data I do not think the subject should be considered to be co-authors.”*
Patients don’t have the skills to be co-authors	12 (16.9)	“They are not scientifically trained” “They may not be fully conversant with the publication process, ethics in scientific publishing”
Patients may be biased	8 (11.3)	“Bias can not be controlled” “Patients are also a part of the conflict of interest.”
Unacceptable in principle	3 (4.2)	“I feel medical journals are for professional publications only” “It is the researcher who is responsible for such activities.”
Other	14 (19.7)	“Only for nonintervention studies” “I have no opinion one way or the other. It is not relevant to the subject matter of our journal”

^a^There were 71 unique statements provided, but some were coded as falling into two categories

When asked specifically about the ICMJE authorship criteria, and whether this should be revised to be more inclusive of patient partners, 35.8% (*N* = 39) indicated it should be revised, 35.8% (*N* = 39) indicated it should not be revised, and 28.4% (*N* = 31) were unsure about a revision. A total of 31 (27.7%) of participants provided an explanation for their response describing why or why not the ICMJE should be revised to consider patient partners in a text box. These explanations were thematically grouped into 7 categories, which are presented in Table 5.

**Table 5:** Thematic grouping of text-based responses describing why/why not the ICMJE should be revised to consider patient partners

Category	N (%)^a^	Example statements
Clearer definition(s) of patient contributions needed	7 (22.6)	“Guidelines should clearly state the terms and conditions for the patient as an author or a partner author.” “Providing clarity would improve editors’ and authors’ confidence to include patients in research.”
Criteria needs to be revised to protect patients	3 (9.7)	“It could take into account the power relations and distributions of patients.”
Depends on certain circumstances	5 (16.1)	“But to limited extent. In a clinical trial role should be limited. In case report, may be role should be more.”
Not a common issue	3 (9.7)	“How common is the issue of patients being involved as authors of scientific papers? If this is relatively rare then possibly mention of this in the guidelines would be sufficient.”
Criteria are complete	3 (9.7)	“I think the criteria look complete from my perspective”
Patients unlikely to meet all criteria	6 (19.4)	“I think the accountability criteria may be challenging for some patients.”
Other	8 (25.8)	“But would need wider discussion in academic community”

^a^There were 31 unique statements provided, but some were coded as falling into two categories

Acceptability of the individual components of the ICMJE authorship criteria by Editors-in-Chief varied. 64.2% (*N* = 70) of Editors-in-Chief felt patient partners could “make a substantial contributions to the conception or design of the work; or the acquisition, analysis, or interpretation of data for the work”. 56.0% (*N* = 61) of Editors-in-Chief felt patient partners could contribute to drafting “the work or revising it critically for important intellectual content”. 67.9% (*N* = 74) of Editors-in-Chief felt a patient partner would likely be able to “give final approval a manuscript to be published”. 42.6% (46) of Editors-in-Chief felt patient partners could be “accountable for all aspects of the work in ensuring that questions related to the accuracy or integrity of any part of the work are appropriately investigated and resolved”. 74.1% (*N* = 80) of Editors-in-Chief did not think patients should be required to have an academic affiliation to published while 16.7% (*N* = 18) and 9.3% (*N* = 10) indicated they should or were unsure, respectively. A total of 24 Editors-in-Chief provided a written response explaining their response to the item about whether patients should be required to have an academic affiliation to publish. Responses were thematically grouped into 5 categories, which are presented in Table 6.

**Table 6:** Thematic grouping of text-based responses describing why patient should/should not be required to have an academic affiliation to publish

Category	N (%)^a^	Example statement(s)
Depends on circumstances	3 (12.5)	*“Highly recommendable but under unusual circumstances e.g highly qualified individual who is a pensioner, exceptions may be allowed.”*
Similar process for patients and researchers	3 (12.5)	*“Just like clinicians don’t necessarily have academic affiliation, neither should patients. Nonetheless, if included they should be fully aware of all research aspects (including meeting authorship criteria)”*
Inappropriate expectation (for affiliation)	12 (50.0)	*“Since their involvement is as a patient or member of the public, I see no reason to require an academic appointment.”*
Moral justification for patient authorship without affiliation	2 (8.3)	*“I am thinking particularly of indigenous, LGBTQ, and other marginalized populations and the issue of epistemic injustice. They must be able to have a voice without being part of an organization/system that may seem oppressive* etc. *to them. This goes back to my point that individuals should be responsible for their contributions and their expertise but not for the expertise of others. The epistemic injustice issue is of particular concern to me.”*
Other	5 (20.8)	*“Up to the PI of the study”* *“The institution where the work originates from is their affiliation”*

^a^There were 24 unique statements provided, but some were coded as falling into two categories

### Editors-in-Chief descriptions of their journal’s operations in relation to patient partnership

Descriptive information about their journal’s operations in relation to patient partners are summarized in Table 7. Key highlights are described here. 93.6% (*N* = 102) of Editors-in-Chief indicated that patients were not involved in any way in the operations of their journal. Among those indicating that patients were involved in their journal, text-based responses (*N* = 10) indicated roles including acting as peer-reviewers, being on the editorial board, or being involved as authors, often in journal sections dedicated to highlighting patients such as patient perspective articles. 1.5% (*N* = 2) of participants indicated patients were always involved in peer review of submitted articles, and 11.7% (*N* = 13) indicated that they always published non-technical summaries of research articles.

**Table 7:** Editors-in-Chief descriptions of their journal’s operations in relation to patient partnership

	N (%)
Does your journal publish non-technical (lay) summaries of research articles? (*N* = 111)	
Yes, always	13 (11.7)
Yes, for some articles	14 (12.6)
No	84 (75.7)
Does your journal involve patients in the peer review of submitted articles? (*N* = 110)	
Always	2 (1.8)
Sometimes	13 (11.8)
Never	95 (86.4)
Are patients involved in any way in the operation of your journal? (*N* = 109)	
Yes	7 (6.4)
No	102 (93.6)
Does your journal specify that it adheres to the ICMJE Criteria for Authorship? (*N* = 111)	
Yes	75 (67.6)
No	17 (15.3)
Unsure	19 (17.1)
Has your journal published a paper that contained a patient or patient partner as author or co-author in the past 12 months? (*N* = 110)	
Yes	16 (14.5)
No	72 (65.6)
Unsure	22 (20.0)
Does your journal have an authorship policy which specifies how patients or patient partners should be considered as authors? (*N* = 110)	
Yes	4 (3.6)
No	98 (89.1)
Unsure	8 (7.3)

67.6% (*N* = 75) of Editors-in-Chief indicated that their journal specifies that it adheres to the ICMJE authorship criteria, 15.3% (*N* = 17) indicated their journal did not adhere to ICMJE authorship criteria, and 17.1% (*N* = 19) of Editors-in-Chief indicting they were unsure if their journals used these criteria. 14.5% (*N* = 16) of Editors-in-Chief indicated their journal had published a paper with a patient or patient partner as an author or co-author in the last 12 months. 3.6% (*N* = 4) of Editors-in-Chief indicated their journal had a policy which specifies how patients or patient partners should be considered as authors. Three of these four Editors-in-Chief provided additional information about what their journal’s patient authorship policy was, of these two stated the policy was the same as or similar to the ICMJE criteria. The third noted their journal encourages patient authorship and described sections in their journal relevant to patients but did not specifically describe a policy.

### Journal type and patient partnership and authorship variables

In our protocol we indicated we would examine differences between responses to the authorship items (items 10–16 in Appendix 2) using t-tests, but given the categorical nature of this data, we conducted Chi-Squared tests. One of these Chi-squared tests was statistically significant: Editors-in-Chief of open access and subscription based journals differed in their responses to the item asking about whether patient partners would likely meet the ICMJE criteria to make “substantial contributions to the conception or design of the work; or the acquisition, analysis, or interpretation of data for the work” (*p* = 0.03,X^2^ = 6.99, df = 2) with more Editors-in-Chief of subscription-based journals indicated ‘yes’ to this item.

## Discussion

Our survey found that while most Editors-in-Chief find it acceptable to list patients and patient partners as authors or co-authors on publications, a large proportion (30.8%) find it unacceptable. If a journal’s Editor-in-Chief does not support patient or patient partner authorship, this is likely to create a barrier to these groups as they look to share their research and obtain appropriate recognition for their contributions. Written responses revealed that some Editors-in-Chief felt patient authors can contribute positively to research and that permitting authorship of contributing patients was important to support equity. Some Editors-in-chief felt authorship was only appropriate if patients met the same standards of involvement as researcher, yet others commented that the likelihood of patients meeting this standard was low since they don’t have the same skills as researchers. Others demonstrated a misunderstanding of patient partnership in research or disagreed in principle, specifying how patients only role is participation in the study or describing concerns about bias of patient participation as authors. This may suggests some Editors-in-Chief misunderstood the context of our questions, perhaps because they do not have exposure to, or training in, patient partnership in research.

Editors-in-Chief were split on whether the ICMJE criteria needed to be updated, with an equal proportion of Editors-in-Chief indicating ‘yes’ and ‘no’ (35.8% each), and the remaining 28.4% indicating ‘unsure’ to this item. Some Editors-in-Chief elaborated on their response indicating that clearer definitions of patient contributions as authors are needed, and that the criteria used need to protect patients. Others noted that the ICMJE criteria were complete, and that patient authorship was not a common “issue”, suggesting ICMJE criteria need not be adjusted. We found variability in Editors-in-Chief perceptions of the likelihood that a patient partner would adhere to each of the authorship four criteria described by the ICMJE. If existing authorship criteria do not appropriately apply to patients this could have implications for how patient partners feel and their perception of inclusiveness within the research team. Given that so few journals appear to have other existing policies to support patient or patient partner authorship (3.6%), this lack of consensus on how to implement the ICMJE authorship criteria for patient partners is highly problematic. Our findings suggest the potential to adapt the ICMJE authorship criteria (this implies research processes are adapting not the patient partners to research processes) to patient partner authors.

There are further nuances in patient partner authorship to consider: 16.7% of Editors-in-Chief indicated that patients should have an academic affiliation to publish and a further 9.3% responded they were ‘unsure’ about this. Text based responses elaborated on these choices: several Editors-in-Chief described how requiring an affiliation is an inappropriate expectation, and others described how there may be moral justification to allow patients to have a voice without being part of “the system”. Others pointed out that this requirement may depend or the situation, or highlighted equality issues, for example, highlighting that since clinicians don’t necessarily need academic affiliations to publish, this process should be extended to patients. We would argue that the contributions of patient partners are unique from that of academic researchers and not meant to duplicate their qualifications but to provide a greater breadth of perspective.. For this reason, we would argue that academic affiliation is unnecessary.. Certainly, some patient partners may have academic affiliations – these may or may not be relevant to the reported research area. If the academic institute to which they are affiliated had no role in the work they are acting on, in our view it would not be appropriate to use their affiliation. However, patient partners may wish to disclose their affiliation transparently in the manuscript dealation section.

Our study had a modest response rate. One challenge in conducting this survey was that many Editor-in-Chief e-mails were not listed on journal websites. This seems like a barrier journals should address to ensure transparency in their operations and ease communication for those wishing to interact with the journal. Future research could build on our findings by gathering more detailed information on Editors-in-Chief perceptions of patient partners as authors. It would also be interesting to more closely examine if there are regional differences in Editor-in-Chiefs perceptions for how patient partner should be involved as authors. In this regard, it may be interesting to consider non-English journals, which were not included in the current study.

## Conclusion

This study provides evidence that the ICMJE authorship criteria might not be best suited to patient partners. We recommend that these criteria be discussed and amended to better reflect the biomedical landscape which includes patients as active and important contributors to research. Revisions to the ICMJE criteria, or elaborated guidance on how to apply the existing criteria to patient partners, should be considered. Such changes ought to be co-designed with patient partners in order to ensure they reflect the community needs more appropriately.

Alternatively, new guidance specific to patient partners could be developed. Recently, the Strategy for Patient-Oriented Research Chronic Pain Network, which is funded by the Canadian Institutes of Health Research, shared a Guidance document on authorship with and acknowledgement of patient partners in patient-oriented research [19]. This document was co-developed by patients and researchers. Conceivably, with consultation of all stakeholders, this process could be replicated to inform journal and publisher policy on patient partner authorship and acknowledgement.

Authorship on academic research papers confers credit to those who conducted and contributed to the research. In some cases, where patients have contributed substantially to the research, they may be relevant to include as authors. Clear policy on journal procedures for handling patient partners as authors is necessary to establish equitable guidance and norms and to recognize their contributions as part of the research team. Failing to capture patient partner contributions when reporting research publications negatively impacts transparency and it does not support the inclusion of patient perspectives systematically into research. Authorship is one of many ways to reward or acknowledge patient partner contributions. We recognize that there may be benefits to patient partners for their contributions, including financial reimbursement of time and expertise, and patient partner preferences for reward or acknowledgement may differ. A qualitative approach, such as an interview study, may help to identify barriers and facilitators to implement appropriate authorship guidance and related patient partner training on patient partner authorship (See for example [20]). This research should be co-designed with patients, researchers and Editors-in-Chief. An important related issue will be the ongoing need to educate Editors-in-Chief and the broader biomedical community about the value and role of patient-partners in the entire research cycle.

## Supplementary Information

**Additional file 1.**

## Data Availability

All study data are publicly available here: https://osf.io/n9g28/.

## Appendix 1

### Recruitment script

Dear <insert Editor-in-Chief’s name>

You are being asked to participate in a research study that we are conducting. Participation is voluntary.

I am part of a team of researchers from the Ottawa Hospital in Canada conducting research to better understand perceptions of patient authorship in medical journals. Patient engagement in research, where patients are partners on the research team and conduct research (in some capacity) alongside researchers, is rapidly growing across the globe. We are interested in examining how patient co-authorship on research publications is understood by medical journal editors and how applicable the ICMJE (International Committee of Medical Journal Editors) criteria for authorship is to patient engagement.

**A random sampling of medical journals was obtained and your contact information was retrieved based on your role as an Editor-in-Chief of < insert journal name here>.**

We are writing to enquire about your willingness to complete an anonymous survey about patient engagement and patient co-authorship. If you are interested in participating, please click the link below to complete the short survey (< 10 min). This link will first redirect you to an online consent form which provides more information about the study and then to the survey. Please do not forward this survey onward.

<insert link here>

By completing the survey, you are providing your consent to participate in the study. I will send two reminder e-mails in 1 and 2 week’s respectively. Your participation would be greatly appreciated. Please do not hesitate to get in touch should you have any questions.

Best wishes,

Kelly Cobey

## Appendix 2

### Survey of editors

*Journal Items*

1. Is your journal fully open access (e.g., all articles are immediately published in a format that makes them free to use and free to build upon to all readers, for example, through Creative Commons licensing)? (Yes/No/Unsure)
2. What is the current impact factor of your journal?
  - < 2.0
  - 2.0–4.9
  - 5.0–8.0
  - 8.0–15.0
  - > 15
  - The journal does not have an impact factor
  - Unsure
3. What type of research does your journal publish?
  - Primarily clinical research
  - Primarily basic or pre-clinical research
  - A combination of both clinical research and pre-clinical research
  - Other, please specify:
4. What area best describes the type of research your journal publishes?
  - General medical journal
  - A research methods medical journal (e.g., systematic reviews journal)
  - A clinical specialty journal (e.g., Cancer research)
  - Other, please specify:
5. Which country is your journal based in? (Drop down)
6. Does your journal publish non-technical (lay) summaries of research articles?
  - Yes, always
  - Yes, for some articles
  - No
7. Does your journal involve patients in the peer review of submitted articles?
  - Always
  - Sometimes
  - Never
8. Are patients involved in any way in the operation of your journal? Yes/No
  - If yes, specify how
9. Does your journal specify that it adheres to the ICMJE Criteria for Authorship?
  - Yes
  - No
  - Unsure

*Editor Items*

10. How long have you served as the Editor-in-Chief of your journal? < 1 year, 1–2 years, 3–4 years, 5–6 years, > 7 years
11. Age (< 25, 26–35; 36–45; 46–55; 56–65; 65+)
12. Gender
  - Male
  - Female
  - Other
  - Prefer not to say

*Authorship Items*

Patient partnership is when patients actively engage with research project teams to help inform the design, conduct, reporting, and translation of a research study.

Patients are defined here according to the Strategy for Patient-Oriented Research (SPOR) definition: “An overarching term inclusive of individuals with personal experience of a health issue and informal caregivers, including family and friends.” (https://cihr-irsc.gc.ca/e/documents/spor_framework-en.pdf)

13. In your view, is it appropriate for patient partners to be authors or co-authors on published biomedical research articles? (yes/no)
  Please specify why/why not:
14. Has your journal published a paper that contained a patient or patient partner as author or co-author in the past 12 months? (yes/no/unsure)
15. Does your journal have an authorship policy which specifies how patients or patient partners should be considered as authors? (yes/no/unsure)
  - If yes, what does the policy say?

The ICMJE recommends that authorship be based on the following 4 criteria:

1. Substantial contributions to the conception or design of the work; or the acquisition, analysis, or interpretation of data for the work; AND
2. Drafting the work or revising it critically for important intellectual content; AND
3. Final approval of the version to be published; AND
4. Agreement to be accountable for all aspects of the work in ensuring that questions related to the accuracy or integrity of any part of the work are appropriately investigated and resolved.
5. In your view, which criteria of the ICMJE authorship criteria would a patient partner be likely to meet?

- Substantial contributions to the conception or design of the work; or the acquisition, analysis, or interpretation of data for the work; (yes/no/unsure)
- Drafting the work or revising it critically for important intellectual content; (yes/no/unsure)
- Final approval of the version to be published; (yes/no/unsure)
- Agreement to be accountable for all aspects of the work in ensuring that questions related to the accuracy or integrity of any part of the work are appropriately investigated and resolved. (yes/no/unsure)

17. In your view, do you think the ICMJE authorship criteria should be revised to be more inclusive to patient partners? (yes/no/unsure); Open text response option to provide any further information.
18. Should patients have to have an academic affiliation to publish as an author/co-author? (yes/no/unsure); Open text response option to provide any further information.

## Abbreviations

BMJ: British Medical Journal
DOAJ: Directory of Open Access Journals
ICMJE: International Committee of Medical Journal Editors
PCORI: Patient-Centered Outcomes Research Institute
SPOR: Society for Patient Oriented Research
