# Supplementary material for: Editors-in-chief perceptions of patients as (co) authors on publications and the acceptability of ICMJE authorship criteria: a cross-sectional survey
Source: Res Involv Engagem. 2021 Jun 14;7:39. doi: 10.1186/s40900-021-00290-1 (PMC8201727; doi:10.1186/s40900-021-00290-1)
Supplement: Supplementary file 1 — Additional file 1. [file 40900_2021_290_MOESM1_ESM.docx]

| Section and topic | Item | Reported on page No |
| --- | --- | --- |
| 1: Aim | Report the aim of PPI in the study | PG 8; …This study was co-designed and produced with a patient partner (LP). The patient partner provided feedback on the protocol indicating the relevance and importance of the topic given their experiences with academic publishing and experiences with research teams. |
| 2: Methods | Provide a clear description of the methods used for PPI in the study | PG 8; …They were involved in the development of the research question, drafting of the study protocol, design of the survey, and with the revision of the manuscript. Their feedback resulted in the addition of survey questions about how patients are included in the operations of journals and journal communications (e.g. plain language summaries). |
| 3: Study results | Outcomes—Report the results of PPI in the study, including both positive and negative outcomes | They were involved in reviewing the study results and identifying new elements such as open-text comment analysis which were reported in the study findings. |
| 4: Discussion and conclusions | Outcomes—Comment on the extent to which PPI influenced the study overall. Describe positive and negative effects | Patient and public involvement contributed to reporting additional findings, context for analysis (e.g. patient perceptions of inclusion on research teams), and validated findings based on their extensive experiences with academic publishing. |
| 5: Reflections/critical perspective | Comment critically on the study, reflecting on the things that went well and those that did not, so others can learn from this experience | The patient partner reports feeling adequately included in the study from the creation of the protocol, survey design, execution, analysis and in the creation of the manuscript. They were kept updated of the various steps of the project and felt like they were able to make a meaningful contribution. |
